# What about Memory, Consciousness, Recall, and Awareness in Anesthesia?

**Published:** 2014-05

**Authors:** Marco Cascella

**Affiliations:** Department of Anesthesia, Endoscopy and Cardiology, Division of Anesthesia, Pain Relief and Intensive Care Unit;National Cancer Institute, Pascale Foundation, Naples, Italy

## Dear Editor,

I read with interest the article by Hadavi  and colleagues rgarding the Evaluation of the Adequacy of General Anesthesia in Cesarean Section by Bispectral Index.^1^

It is fair to say that at the moment the Bispectral Index (BIS) does not offer guarantees for awareness  prevention and as reported by Avidan, the use of the Bispectral Index could give anesthesiologists a false sense of security that if they keep the measurement between 40 and 60, they will prevent anesthesia A.^2^Why do we continue to use the BIS ? It simply guides us in saving drugs and facilitating recovery from anesthesia.^3^

Your statements regarding benzodiazepines are correct, because, although the American Society of Anesthesiologists (ASA) recommends their use in select cases,^4^ their effects on brain connectivity,^5^ and therefore the impact on consciousness as well as their role in anterograde and retrograde memory^6^are well documented.

However, I have to make some clarifications. I noted an inaccuracy in the statistics. The incidence of awareness has been reported at 0.10%-0.20%, therefore a court of 60 patients was inadequate to reach a precise conclusion.

I observed confusion in the terminology that has pertained to explicit memory, consciousness, recall and awareness. Explicit memory is a type of long-term memory that relates to facts or events secondary to the consolidation time dependent. The terms recall and awareness can be used interchangeably because the exact definition of intraoperative awareness is the presence of both consciousness and explicit memory with recall of surgical events. With this definition, awareness, and therefore, recall, shall provide power for a moment of consciousness. 

In your study, patients were interviewed up to 24 hours after surgery. There have been reports of awareness  up to one week after anesthesia, although they are less likely.

Post-traumatic stress syndrome (PTSD) is a well-defined syndrome (DSM-V) that constitutes a clear precipitating event, which in our case is awareness. The incidence of PTSD post-awareness is unclear and ranges from 2% to 71% of awareness cases. Thus, PTSD cannot exist post-awareness without awareness.

In the near future, there will be new technologies available that monitor the depth of anesthesia based on the theory of brain connectivity by Tononi et al.^7^^,^^8^ It is my hope you can use these technologies to report your studies in the literature.

## References

[1] Hadavi SM, Allahyary E, Asadi S (2013). Evaluation of the Adequacy of General Anesthesia in Cesarean Section by Bispectral Index. Iran J Med Sci.

[2] Avidan MS, Zhang L, Burnside BA, Finkel KJ, Searleman AC, Selvidge JA (2008). Anesthesia Awareness and the Bispectral Index. N Engl J Med.

[3] Shepherd J, Jones J, Frampton G, Bryant J, Baxter L, Cooper K (2013). Clinical effectiveness and cost-effectiveness of depth of anaesthesia monitoring (E-Entropy, Bispectral Index and Narcotrend): a systematic review and economic evaluation. Health Technol Assess.

[4] American Society (2006). Practice advisory for intraoperative awareness and brain function monitoring: a report by the american society of anesthesiologists task force on intraoperative awareness. Anesthesiology.

[5] Veselis RA, Reinsel RA, Feshchenko VA, Wroński M (1997). The comparative amnestic effects of midazolam, propofol, thiopental, and fentanyl at equisedative concentrations. Anesthesiology.

[6] Timić T, Joksimović S, Milić M, Divljaković J, Batinić B, Savić MM (2013). Midazolam impairs acquisition and retrieval, but not consolidation of reference memory in the Morris water maze. Behav Brain Res.

[7] Tononi G (2004). An information integration theory of consciousness. BMC Neurosci.

[8] Alkire MT, Hudetz AG, Tononi G (2008). Consciousness and Anesthesia. Science.

